# Low-Velocity Impact Behavior of Foam Core Sandwich Panels with Inter-Ply and Intra-Ply Carbon/Kevlar/Epoxy Hybrid Face Sheets

**DOI:** 10.3390/polym14051060

**Published:** 2022-03-07

**Authors:** Stanley Samlal, R. Santhanakrishnan

**Affiliations:** 1 Structural Impact and Crash Simulation Centre (SIMCRASH), School of Aeronautical Sciences, Hindustan Institute of Technology and Science, Chennai 603103, India; 2 School of Aeronautical Sciences, Hindustan Institute of Technology and Science, Chennai 603103, India; santhanak@hindustanuniv.ac.in

**Keywords:** low-velocity impact, hybrid epoxy composite, damage tolerance, carbon, kevlar, inter-ply, intra-ply

## Abstract

Sandwich composites are extensively employed in a variety of applications because their bending stiffness affords a greater advantage than composite materials. However, the aspect limiting the application of the sandwich material is its poor impact resistance. Therefore, understanding the impact properties of the sandwich structure will determine the ways in which it can be used under the conditions of impact loading. Sandwich panels with different combinations of carbon/Kevlar woven monolithic face sheets, inter-ply face sheets and intra-ply face sheets were fabricated, using the vacuum-assisted resin transfer process. Instrumented low-velocity impact tests were performed using different energy levels of 5 J, 10 J, 20 J, 30 J and 40 J on a variety of samples and the results were assessed. The damage caused by the modes of failure in the sandwich structure include fiber breakage, matrix cracking, foam cracking and debonding. In sandwich panels with thin face sheets, the maximum peak load was achieved for the inter-ply hybrid foam core sandwich panel in which Kevlar was present towards the outer surface and carbon in the inner surface of the face sheet. At an impact energy of 40 J, the maximum peak load for the inter-ply hybrid foam core sandwich panel was 31.57% higher than for the sandwich structure in which carbon is towards the outer surface and Kevlar is in the inner surface of the face sheet. The intra-ply hybrid foam core sandwich panel subjected to 40 J impact energy demonstrated a 13.17% higher maximum peak load compared to the carbon monolithic face sheet sandwich panel. The experimental measurements and numerical predictions are in close agreement.

## 1. Introduction

Foam core sandwich composites are used in industries such as aerospace, automobile, wind turbines, marine functional materials and even in home appliances because of their high strength to weight ratio, good buckling resistance and tailorable design which remains dimensionally stable across a wide temperature range [1,2,3,4,5]. Sandwich foam core structures have distinct advantages which make them the preferred choice for numerous applications involving bending, buckling and energy absorption applications such as dynamic loading, high- and low-velocity impacts, blasts, crashes, etc. [6]. While sandwich composites are designed to resist bending and buckling loads, they also demonstrate excellent energy absorption capabilities [7,8]. Sandwich structures are extensively used in aerospace applications including Unmanned Arial Vehicles (UAV), wind turbine blades and marine structures which are employed extensively under adverse conditions, including impact loads [9].

Impact failure is a common problem that affects the strength and structural integrity of sandwich composites [10,11]. This issue is usually caused by the degradation of energy absorption competence and structural stiffness. The sandwich structure suffers major damage from the impact load, and the damage progressively increases with repeated loading [12,13]. Therefore, hybrid composites are designed and developed to improve different properties such as impact resistance, thermal conductivity, dielectric breakdown strength, flexural and environmental degradation resistance [14,15,16].

A sandwich composite is a type of lightweight structure that has high specific strength and stiffness. It is also suitable for high in-plane and flexural stiffness. Impact applications are limited in sandwich composites due to the absence of good resistance to localized loading. Different types of core materials are developed and used in the construction of sandwich composites. The core should be a low-cost, lightweight material, with the ability to transmit the shear forces generated by face sheets to the other components of the sandwich [17]. High strength metallic face sheets with a honeycomb core are not ideal for the weight-saving of the sandwich panels [18]. The strong and stiff bonding between the core material and face sheets is necessary to enable the sandwich to bear high loads [19]. Kevlar fibers can improve the impact damage tolerance of the composite materials when used together with carbon fibers [20,21,22,23,24,25]. The sandwich construction is composed of two face sheets, separated by a low-density foam core. In hybrid fiber sandwich structures, two or more types of fibers are used in the face sheets to improve the monolithic face sheet properties [25,26,27,28]. The impact and the failure mechanism for the sandwich structure is a very complex mechanism and continues to remain an active topic under discussion by many researchers [29,30,31,32,33,34,35]. Enhancement of the impact properties of the foam sandwich panels through stitching, Z-pinning, tufting and also by implementing changes in the density of the foam core has been investigated by researchers [36,37,38,39,40]. Fiber-reinforced polymer composites are an excellent substitute for face sheets, more than traditional metallic face sheets owing to their ratios of good stiffness to weight, and strength to weight. Even with low-velocity impact, the minor impact can still induce significant damage to structures [41]. Such types of damage can severely weaken the strength and stiffness of a structure. Thus, it is critical to consider the effect of low-velocity impacts on the carbon/Kevlar-epoxy hybrid face sheets with a foam core. With reference to this, Gustin et al. studied the effect of low-velocity impact using different incident face sheet combinations of the Kevlar/carbon foam core sandwich panels [42]. The unidirectional fiber (UDF) face sheet skins are the most commonly available type of face sheet but are very susceptible to impact damage because of the low transverse tensile strength [43]. The bidirectional woven fabric layers used in the manufacturing of major components impact damage tolerance of the unidirectional fiber (UDF)-reinforced sandwich skins is lower than it is for the woven fabric (WF) because of their low transverse tensile strength, better flexibility and ease of the fabrication process. Due to the high specific stiffness and strength, woven carbon fabrics continue to be frequently used in several applications such as aircraft structures, aerospace materials, vehicles and sports gear. The bidirectional woven fabric layers are used in different stacking and weaving sequences to fabricate the hybrid sandwich composites. The different types of sandwich composites made using these combinations include the monolithic face sheets, inter-ply and intra-ply hybrid sandwich composites [44]. Hybridization of face sheets in sandwich composites yields better impact resistance to the structure than the single fiber composite phase. This also permits the use of such types of hybrid composites for specific applications to assemble the exact requirement of the structure and tailor it for that specific application. Recently, attention has been drawn to the low-velocity impact on sandwich structures, and therefore, impact tests have been conducted on sandwich structures with different core and face sheet materials [45,46,47,48]. 

This paper investigates the foam core sandwich structure with different combinations of the hybrid face sheets on both the incident (primary) and the secondary faces with a variety of hybrid combinations. The carbon woven face sheet [C], Kevlar woven face sheet [K], inter-ply face sheet [K/C] with Kevlar towards the outer surface of the sandwich, the inter-ply face sheet [C/K] with carbon towards the outer surface of the sandwich and intra-ply carbon/Kevlar face sheet [H] are the face sheet combinations used to fabricate the different combinations of the foam core sandwich panels. The hybrid face sheet sandwich panel with Divinycell-‘H’ 80 polyvinyl chloride (PVC) closed core (Foam core) was fabricated using the VARTM technique. Sandwich panels were fabricated using the VARTM, with five different combinations of monolithic face sheets, namely [C_2_/Foam core/C_2_]; [K_2_/Foam core/K_2_]; the inter-ply face sheets [C/K/Foam core/K/C]; [K/C/Foam core/C/K]; and intra-ply face sheets [H_2_/Foam core/H_2_]. Specimens with the dimensions of 60 mm × 60 mm were prepared from the panel using the water jet cutting technique in a manner that excluded all residual stress post the cutting process. The low velocity energy impact tests are necessary to understand the impact response of foam core sandwich panels. Impact responses of the panels subjected to different energy levels were obtained and load-time graphs were quantified together with the peak load and depth of indentation. Damage to each specimen was studied through visual observations, and an ultrasound C-Scan and SEM techniques were employed to assess the degree of damage, with different impact energies. Thus, the objective of this study is to develop an experimental framework with which the dynamic response of the foam core sandwich panels can be examined when they are subjected to low-velocity impact tests on hybrid face sheets. The results will help in identifying the failure modes and developing effective solutions in the design of hybrid sandwich panels. These types of sandwich structures can be employed in applications such as UAV’s and marine structures, where the through-thickness transverse depth of the structure is low and both the face sheets of the panel are exposed to the threats of potential impacts. 

## 2. Materials and Methods

The core material selected for this research work was Divinycell closed—cell ‘H’ marked foam core with a density of 80 kg/m^3^ and cross-sectional thickness of 10 ± 0.10 mm manufactured by Diab International. The characteristics of Divinycell- ‘H’ foam core include resistance to fatigue, impact resistance, strength to weight ratio and exceptional cost-efficiency. The Divinycell closed cell foam core is known to be very durable and resilient. In Table 1 are listed the properties of the Divinycell- ‘H’ foam core. Carbon, Kevlar and intra-ply carbon/Kevlar woven fabrics were used as the face sheets in the fabrication of the foam core sandwich panels. The foam core was flanked by two layers of woven fabric on either side. The properties of the woven fabrics used to prepare the panels are given in Table 2. Five different combinations including the monolithic face sheet sandwich panel, the inter-ply sandwich panel and the intra-ply sandwich panel were fabricated using different stacking sequences of the carbon, Kevlar and hybrid layers. The [C_2_/Foam core/C_2_]; [K_2_/Foam core/K_2_]; [C/K/Foam core/K/C]; [K/C/Foam core/C/K]; and [H_2_/Foam core/H_2_] were prepared using VARTM. Exceptional care was taken to ensure that the accurate quantity of resin was added through the resin inlet pipe during the vacuum infusion process. The peel ply was applied carefully during the fabrication process because the wrinkling of the peel ply will affect the surface finish of the fabricated panels. The panels were fabricated using a low viscous, clear, epoxy liquid resin, composed of customized Bisphenol— ‘A’ utilizing the aromatic glycidyl ether called Araldite^®^ GY 257, manufactured by Huntsman Corporation and the properties of which are tabulated in Table 3. The hardener used in the fabrication process was C-2693. The epoxy value of the Araldite^®^ GY 257 resin system is in the 5.15–5.45 eq/Kg range, with the resin viscosity between 525–725 mPa.s. at 25 °C. Low viscosity and complete crystallization resistance are the chief characteristics of the Araldite^®^ GY 257 epoxy resin, which makes it perfect for the VARTM process. The resin and hardener were uniformly mixed in the ratio of 100:45 by percentage weight. Poly-amidoamines or polyamines were used in tandem with this resin system to ensure that curing occurs in a shorter time range. The properties of the Divinycell-‘H’-80 foam, resin and hardener were supplied by the manufacturers. Specimens were cut to the dimension of 60 mm × 60 mm using the water jet cutting technique for a drop weight impact test. The water jet cutting method was employed to ensure that no residual stresses were present in the specimen and to guarantee that no heat was generated during the cutting process which would alter the properties of the manufactured specimens. 

### 2.1. Panel Fabrication

**Figure 1:**
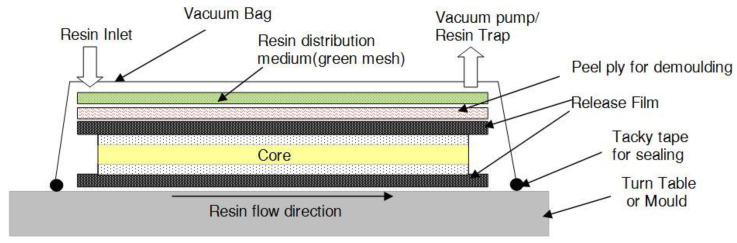
Graphic representation of Vacuum-Assisted Resin Transfer Molding (VARTM) fabrication process for foam core sandwich panel.

The sandwich panels of the dimension 300 mm × 300 mm were fabricated by employing the VARTM process. The VARTM technique is primarily used for the fabrication of defense equipment, military containers and marine equipment, including buoyant mobile structures. Various layers of fiber mats are stacked one directly above the other and impregnated with a resin system utilizing vacuum pressure. The VARTM displays significant advantages over conventional Resin Transfer Molding (RTM) by avoiding the operating expenses related to metal tooling, reducing the volatility and ensuring low working pressure. The VARTM method is completed by a sequential process, which begins with the lay-up of the fabric and sandwich core as dry stack material on a turntable which is used as a mold. The release film, peel ply and the resin distribution medium are positioned in the appropriate spaces as shown in Figure 1. The woven fabric is cut to the dimension of 300 mm × 300 mm. Then, the first layer is placed down on the turntable and the next layer is put over the first, cut to the same dimension. Subsequently, a foam core of the identical size is placed on the two fabric layers, and the sandwich structure is completed by placing the two layers of fabric pieces over the foam core, thus forming the dry stack for each panel. The lay-up of the fiber reinforcement material used in the facing sheets was done in accordance with the different stacking sequences that are listed in Table 4. The dry stack was placed inside a vacuum bag and sealed using tacky tape, and the air was removed utilizing the vacuum pump attached to one side of the closed system. The next step was to initiate the impregnation of the dry stack with the resin hardener mixture from a peripheral tank, employing vacuum pressure. The impregnation of the panel was sustained continually until the entire panel was completely wet. The low pressure of −101.3 kPa was maintained till the entire panel was thoroughly soaked with the resin system and then clamped to sustain the pressure inside the vacuum chamber. The dwell time of the impregnated stack was accomplished, and the panel was kept undisturbed for 24 h of curing time prior to the de-molding of the panel from the turntable and exposing it to the conditions of the ambient state. The Divinycell-‘H’ foam core was prepared using perforations for the easy flow of the resin, before the VARTM process. The perforations ensured that the resin could flow from one side of the foam core to the other, and the grooves prepared ensured the ease of the resin flow through the interfaces between the face sheet and the foam core. Each process was followed meticulously to fabricate the sandwich panel without any flaws or inclusions. The release film, in contact with the face sheet fabric, was used to detach the sandwich from the resin hardener supply medium. This also enabled the release of the trapped gases or unstable compounds, responsible for creating the flaws in the sandwich panel, thereby increasing the panel strength. Peel ply is the woven material, with superior heat resistance, used for covering the dry stacked materials on the molding turntable. This was employed to ensure that the surface finish of the fabricated sandwich panel was excellent; besides this, it also guaranteed that the surface of the sandwich panel was uncontaminated and untainted. The resin distribution medium, a non-woven type structure soaked up the entire excess resin hardener mixture, allowing the trapped gases or unstable compounds of a volatile nature to be released, during the dwell and cure procedure. A resin catch pot was used prior to employing a vacuum pump, to ensure that ample time was available to culminate the process, also that the resin hardener mixture did not contact the vacuum pump. The fabricated sandwich panels were removed from the turntable and machined to the ASTM specification given for the drop weight impact testing.

### 2.2. Low-Velocity Impact Tests

**Figure 2:**
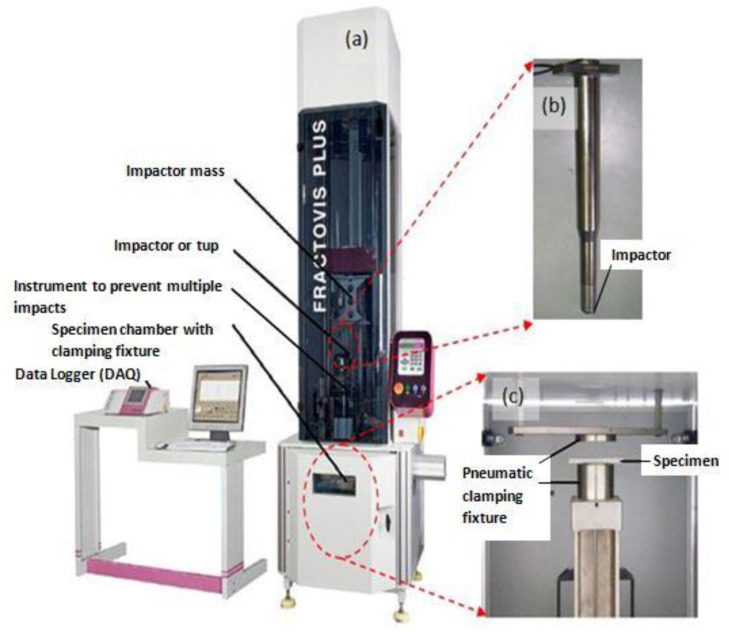
Low-velocity drop weight impact test machine with the data acquisition system (DAQ) and clamping fixture. (**a**) Drop weight machine. (**b**) Impactor of 19.9 mm diameter. (**c**) Pneumatic clamping fixture.

**Figure 3:**
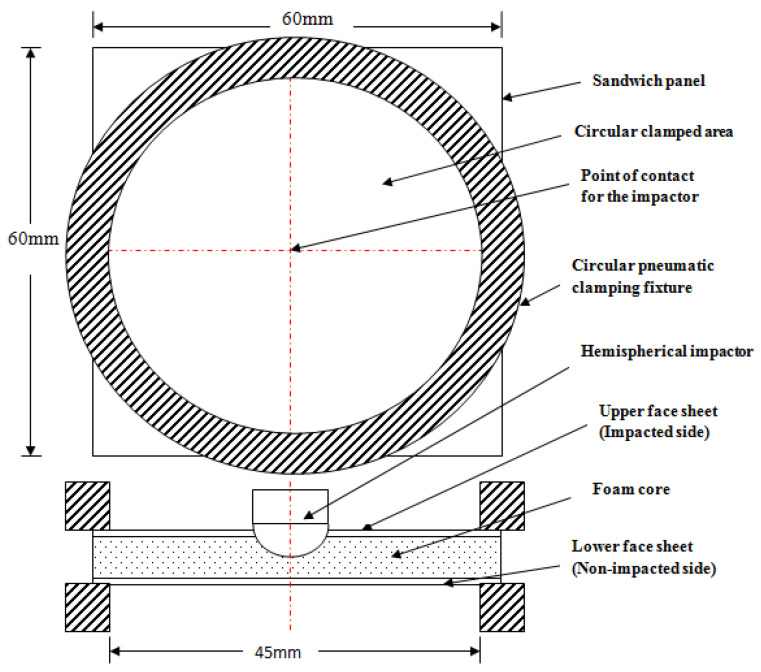
Line diagram illustrating pneumatic circular clamp dimensions of the drop weight impact test machine with the specimen [52].

**Figure 4:**
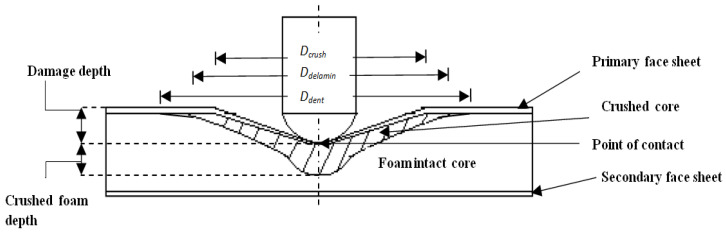
Illustration of foam core sandwich impacted with hemispherical impactor displaying the failure of impacted (primary) face sheet and core crushing.

**Figure 5:**
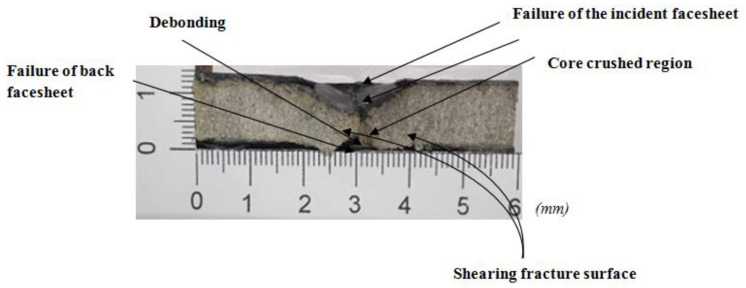
Photographic example of through-the-depth local indentation of the foam core sandwich panel under low-velocity impact after the three stages of the penetration progression: (i) failure of incident face sheet, (ii) core crushing and shearing failure, (iii) failure of the back face sheet.

**Figure 6:**
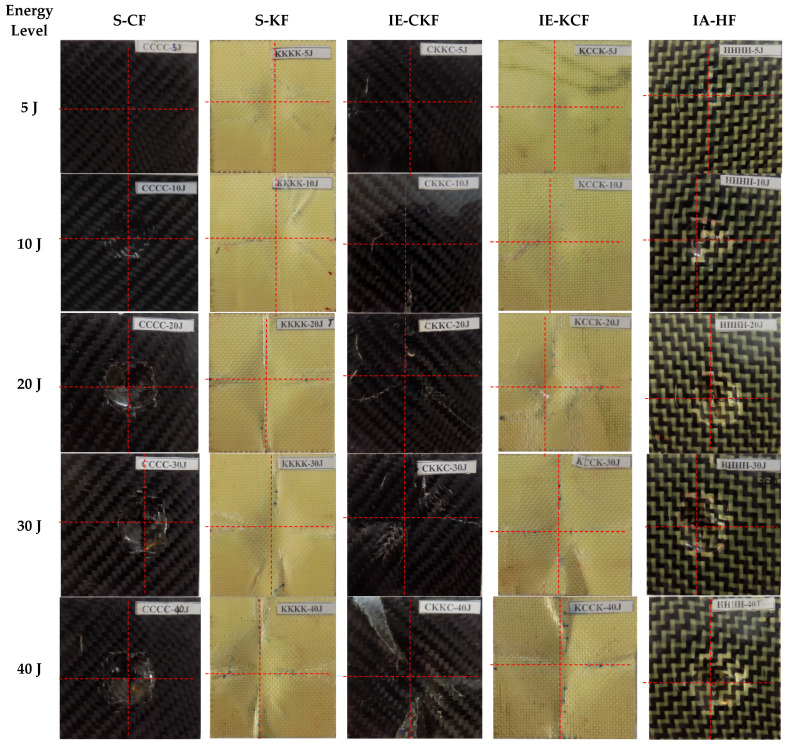
Impacted face sheet photographic images of samples impacted with different energy levels.

**Figure 7:**
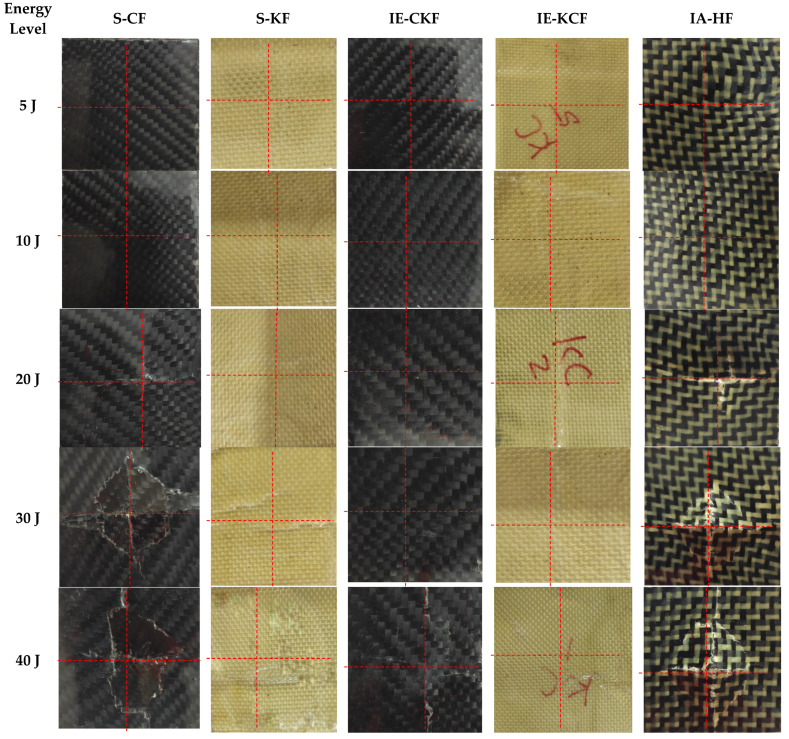
Secondary face sheet photographic images of samples impacted with different energy levels.

**Figure 8:**
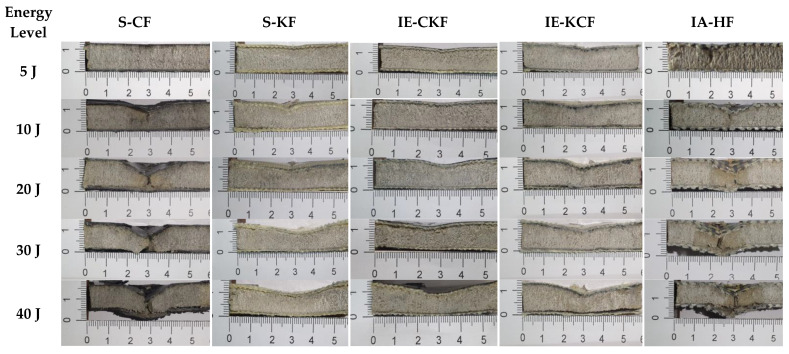
Transverse damage morphology of various foam core sandwich specimens after impact with hemispherical impactor shows the residual indentation, delamination, debonding, core crushing.

The low-velocity impact assessments were conducted on all the foam core sandwich panels with a fully automated Ceast Fractovis-Plus drop weight impact testing operational instrument, coupled directly to a Data Acquisition System (DAQ). The climate control system was attached to the specimen chamber. Maintaining the standard ambient temperature of 25 °C, consistent tests were conducted. The impactor assembly houses the frame, force sensor and contact hemispherical tup. To clamp the specimen prior to impact, a pneumatic fastening system was adopted and meticulous care was taken to ensure that the specimens were not damaged during mounting on the clamps. For all the tests, ASTM standard (refer ASTM D5628 FE) [52] was used. In fact, Figure 2a displays the detailed picture of the drop weight impact instrument with the impactor and pneumatic fastening system. The manufactured test specimen, 60 mm × 60 mm in dimension, was placed between the pneumatic clamping systems. Impact point prediction was performed using laser guides to prevent any misalignment of the specimen. Special care was taken to align the specimen with the line of impact. Each specimen was aligned with the impactor falling line and clamped with a circular clamped area of 1590.43 mm^2^, corresponding to an exposed circular diameter of 4.5 mm. The hemispherical impactor diameter 19.9 mm, as shown in Figure 2b. The specimen dimensions and fixture clamping area are revealed in Figure 3. The potential energy of the falling impactor can be calculated, using the equation,

(1) $$PE_{h}=m_{i}*g*h_{i}$$

where *PE_h_* is the potential energy of the impactor with respect to its height, $m_{i}$ is the total mass of the impactor, *g* is the acceleration due to gravity and $h_{i}$ is the height to which the impactor is raised from the specimen. The velocity of impact can also be calculated by the formula,

(2) $$v_{i}=\sqrt{\left(2*E\right)/m_{i}}$$

where $v_{i}$ is the velocity of impact, *E* is the kinetic energy and $m_{i}$ is the total mass of the impactor (tup and the falling weight impactor assembly) used to apply the impact energy on the sandwich panel. The change from the potential energy to the kinetic energy was used as the input impact energy. For all the tests, the weight of the impactor assembly was maintained constant at 3.19 kg. The input was given to the drop weight impact machine to change the height of the impactor to 159.3 mm, 318.6 mm, 637.1 mm, 955.7 mm and 1274.2 mm equivalent to the energy levels of 5 J, 10 J, 20 J, 30 J and 40 J, respectively. The impact velocities equivalent to the heights were also found to be 1.78 m/s, 2.5 m/s, 3.55 m/s, 4.34 m/s and 5.01 m/s, corresponding to 5 J, 10 J, 20 J, 30 J and 40 J, respectively. For every energy level, three samples of each configuration were tested for low-velocity impact. The load-time (*L-t*) history of all the specimens was quantified, the maximum load, absorbed energy and the primary and secondary peaks with the sudden load variation acted as pointers of damage initiation and damage progression in the specimens. The load-time (*L-t*) curve of each impact event was obtained by the CEAST-Data Logger; the DAS-8000, automated strikers and dedicated software were used to process the data and provide the automated input for the low-velocity impact. The D-8-EXT-WIN-CEAST software was used with FRACTOVIS PLUS for more rapid processing of the data acquired from each test, as well as to perform the instrumented tests and analysis. Line diagram and photographic illustration of through-the-depth local indentation of the foam core sandwich panel under low-velocity impact and after different stages of penetration sequence are shown, correspondingly, in Figure 4 and Figure 5. Impacted face sheet and secondary face sheet photographic images of the samples, impacted with different energy levels, are presented, respectively, in Figure 6 and Figure 7. Through-the-thickness damage morphologies of the different foam core sandwich specimens after impact with the hemispherical impactor are displayed in Figure 8. After the drop weight impact test was completed, the specimens were subjected to post-test analysis by visual inspection, depth/damage area measurements, C-Scan and SEM scans.

### 2.3. Impact Depth Measurement 

**Figure 9:**
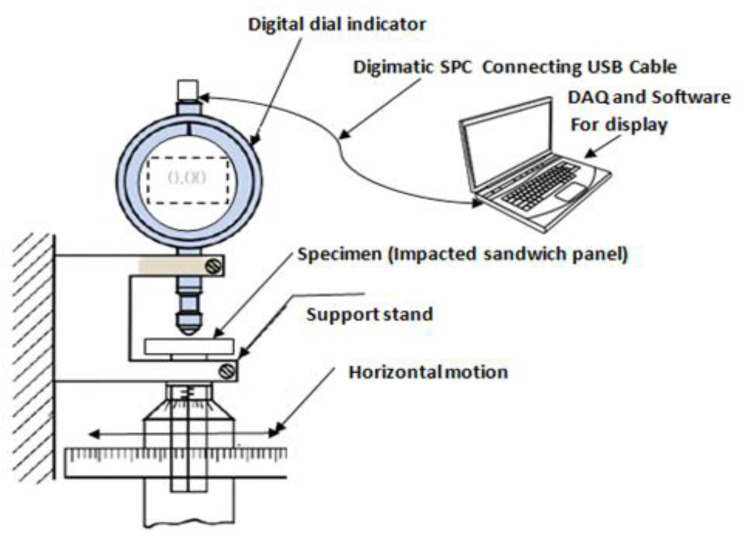
Line diagram depicting the process of determining the incident face sheet residual depth high-precision micrometer gauge after impact.

The Mitutoyo 543-791 high-precision micrometer gauge was used to determine the depth of the impact dent, for each specimen. While the dial gauge has a range of 0.0 to 12.70 mm, a resolution of 0.02 mm was employed to obtain the depth measurement of the incident face sheet. The dial gauge was mounted on a guide rail for horizontal motion over the impacted specimens. After calibration, the reading was set to zero, for each sample, and horizontal motion was provided to the specimen to capture the impact depth of the incident face sheet. The Digimatic-SPC USB cable was used to connect the measuring device to the computer which was used as a Data Acquisition System (DAQ) and the depth was recorded using the dedicated software. The residual damage depth of the top face sheets corresponding to the lengthwise distance of all the sandwich panels was measured with the micrometer gauge. The length wise distance of the impacted samples was measured in multiple perpendicular directions to minimize the error in the measurements. The depth measurements were superimposed for the different energy levels, to display the variations in the residual depths. The residual dent depth graphs showed an increase in the maximum depth corresponding to the rise in the impact energy. The process of depth measurement was done for all impacted specimens with different face sheet. In Figure 9, the line diagram explaining the process of determining the incident face sheet residual depth using the micrometer gauge after impact, is evident.

### 2.4. Damage Evaluation by Immersion Ultrasonic Testing

**Figure 10:**
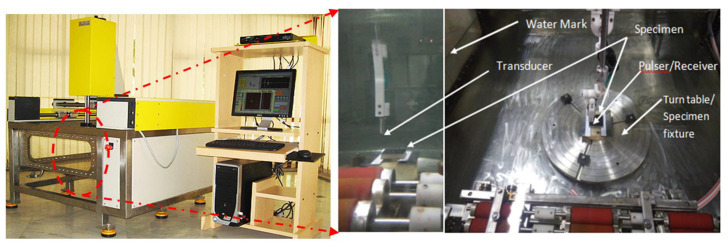
Ultrasonic immersion C-Scan testing system showing the water immersion tank with specimen turn table.

The ultrasonic water immersion C-Scan technique was employed to determine the damage to the specimens. The Dhvani-SHRUTI-011, India scanning high resolution ultrasonic testing machine was used to scan the samples after impact. According to the requirements, to assess the damaged area on the face sheets, the front wall echo was taken as the reference signal. The amplitude of the front wall echo was kept minimal at the damaged area in comparison to the rest of the sample, where the maximum amplitude was detected. The C-Scan images were produced by monitoring the front wall echo. The damaged area was marked on the C-Scan images, with dimensions, together with the scan and index axis. Using the pulse voltage of 153 V for the C-Scan and the transducer frequency of 15 MHz, a probe was employed, having a focal length of 50 mm. The scan resolution and index resolution used in the study was 0.10 mm. The ultrasonic immersion C-Scan testing system used for assessment of damage, is shown in Figure 10. The impacted specimen was immersed in the specimen tank using a suitable fixture with an automatic specimen leveling technique. The ultrasonic transducer probe preset on a computer-controlled three-axis servo motor-powered framework was used to perform the layer-by-layer study.

### 2.5. Microscopic Scan of Fractured Surface

After the drop weight tests were completed, the characteristic Scanning Electron Microscopy (SEM) was done on the sandwich specimens to assess the morphological microstructure with the COXEM model CX-200TA, Korea. The SEM specimens were cut out from the impacted region of the sandwich panels; interpretation was done paying attention to the stacking sequence of the carbon, Kevlar, and hybrid layers, respectively. The foam core crushing phenomenon was clearly observable in the area of impact. The different types of failures, which include of matrix cracking, fiber breakage, fiber-matrix debonding, debonding of the core and face sheets, and core crushing has been comprehensively investigated through the SEM scan. Pictures of the characteristic SEM microstructure in the impact section of the various hybrid sandwich specimens were studied in depth to establish a connection with the different microstructural failures.

## 3. Numerical Modelling

**Figure 11:**
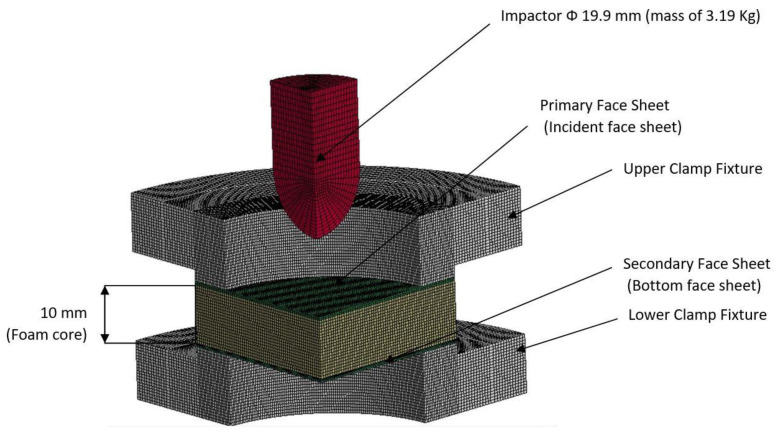
The FE model of foam core sandwich panel with the circular clamp fixture and impactor.

To predict the impact response of the sandwich panels, LS-DYNA Finite Element (FE) models were created. Quarter models were developed for numerical simulation because of the symmetry, and symmetric boundary conditions were used on the planes of symmetry. Figure 11 depicts the typical FE model of a foam core sandwich panel with mesh features. To replicate the experimental conditions, the fixtures were modelled, and to imitate the clamped condition, the plate and fixture arrangement were constrained in all degrees of freedom. The aspect ratio, with element dimensions of 0.5 mm, was employed throughout the study for foam core. To avoid spurious modes, hourglass control was utilized, and hourglass energy was controlled within 10% of the peak internal energy value. The eroding surface to surface contact option was used to make contact between the panel and the impactor. To mimic bonding between multiple surfaces, tie break constraints were used. Because the contact time between the impactor and the sandwich plate was so short, the effect of friction between them was ignored. If the time step was reduced to 60% of its initial value, excessive element distortions and shorter time frame steps (induced by thinning of the element) were prevented by eroding elements. A hemispherical impactor with a mass of 3.19 kg and pneumatic clamps was modelled with the 8 noded brick elements and modelled as a rigid body using steel properties (Youngs modulus of 200 GPa, density of 7860 kg/m^3^, and Poissons ratio of 0.3). Enhanced composite damage material with Chang-Chang failure criteria was used to simulate various composite face sheets (*ENHANCED COMPOSITE DAMAGE, MAT 54). Material Types 54 are improved variants of the composite models. Various sorts of failure can be supplied as an option, based on Chang-Chang or Tsai-Wu recommendations. In addition, specific precautions have been implemented in the event of compression failure. Material characteristics for carbon [53,54], Kevlar [55], and intra-ply epoxy [56] composite face sheets were gathered from the literature as inputs. The homogenised honeycomb model was used to simulate the core materials (H80) (*MAT HONEYCOMB, MAT 26). Orthotropic foams may be described in this constitutive model by establishing various compression stress–strain curves in different orientations. The connection between the stress components is not taken into account in the homogenised honeycomb model. The data of material properties and response curves used to model H80 foam were obtained from the literature [57]. Maximum strain failure criteria were always applied for H80 foam. The (*MAT ADD EROSION) option was employed with the tensile volumetric strain criteria. A detailed mesh convergence investigation was carried out along the longitudinal and transverse directions of the sandwich panel using several sets of elements. There are 11,08,048 solid elements and 12,42,431 nodes in the finite element model used to study the low-velocity impact. All computations were performed on a high-performance computing machine.

## 4. Results and Discussion

This section summarizes and discusses the main findings of the present work, done using the low-velocity impacts on the monolithic and hybrid face sheet sandwich panels. Different models of damage are revealed in the visual inspection of the impacted face sheet in Figure 6 and Figure 7. The explicit reaction force exerted by the material sample in opposition to the force applied by the impactor is termed contact force or load. When low impact energies are applied on the specimen, the load-time (*L-t*) history is in the form of a parabolic curve and the limit contact force increases with the rise in the impactor force for all the different configurations of the panels subjected to the low-velocity impact. The post-impact inspection of the panels reveals the damage caused by the different modes in the face sheets and foam. The failure modes are investigated by visual inspection, and the C-Scan and SEM scan methods. The results of the inspections give evidence of fiber breakage, matrix failure, debonding of the core and face sheet and core crushing as the primary causes of the damage.

### 4.1. Contact Load with Time History of Combinations of Hybrid Face Sheet Specimens

**Figure 12:**
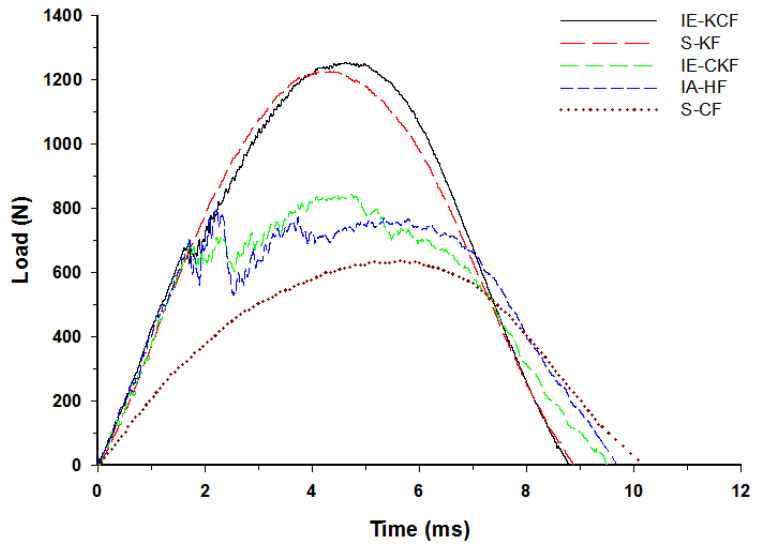
Load-time history of different foam core sandwich specimens at 5 J.

**Figure 13:**
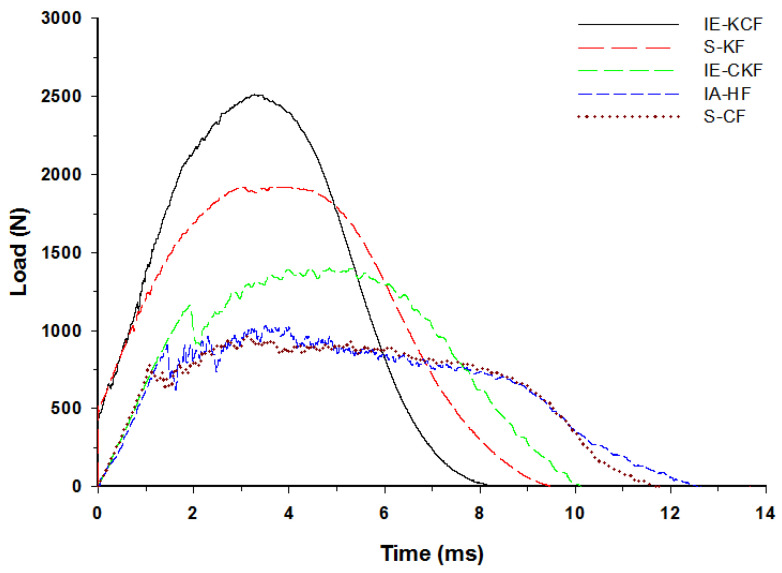
Load-time history of different foam core sandwich specimens at 10 J.

**Figure 14:**
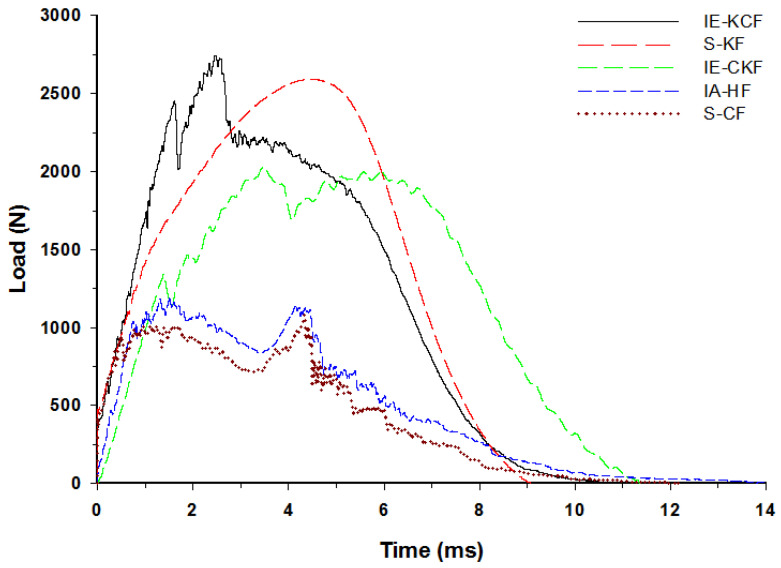
Load-time history of different foam core sandwich specimens at 20 J.

**Figure 15:**
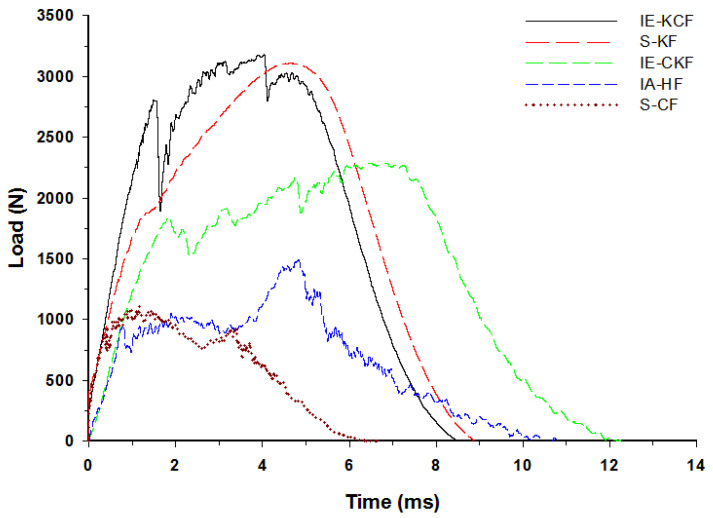
Load-time history of different foam core sandwich specimens at 30 J.

**Figure 16:**
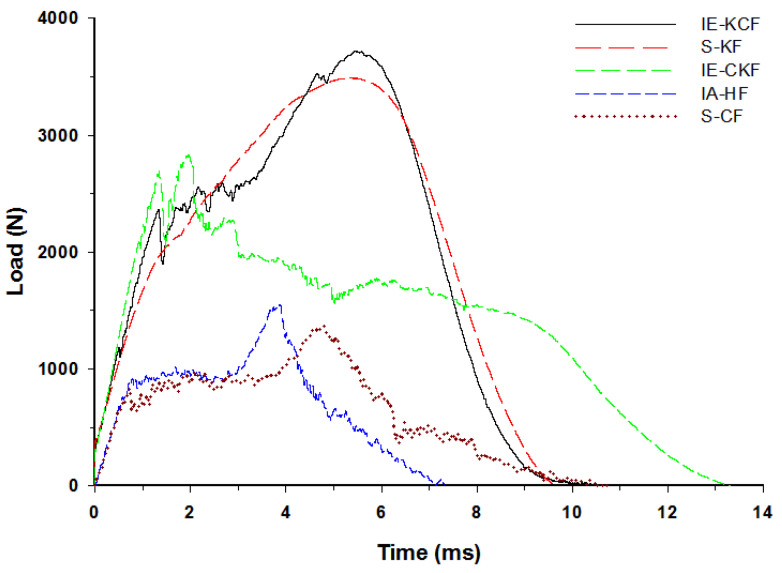
Load-time history of different foam core sandwich specimens at 40 J.

The typical contact load-time (*L-t*) graph of the sandwich panel can be divided into three distinct regions predicated and interpreted namely a primary peak, followed by a plateau region and a secondary peak [30]. The primary apex of the load-time (*L-t*) graph shows the perforation of the impacted side face sheet and the plateau region with the slow increase in force corresponds to the core crushing process; the secondary peak shows perforation of the secondary face sheet, which culminates in the complete perforation of the sandwich panels. Visual examination was made for the higher energy impacts; for lower energy impacts these regions were less distinct [37]. With each increment in the impact energy, a corresponding increment was noted in the reaction force applied by the specimen on the impactor. In fact, Figure 12, Figure 13, Figure 14, Figure 15 and Figure 16 display the characteristic load-time (*L-t*) graphs for the impacted combinations of the foam core sandwich panels, at the different impact energy levels of 5 J, 10 J, 20 J, 30 J and 40 J, respectively.

Figure 11 depicts the typical load-time (*L-t*) graphs for [C_2_/Foam core/C_2_]; [K_2_/Foam core/K_2_]; [C/K/Foam core/K/C]; [K/C/Foam core/C/K]; and the [H_2_/Foam core/H_2_] combinations of the foam core sandwich panels, subjected to an impact load (contact force) of 5 J. The maximum load can be defined as the highest reaction force exerted on the impactor by the sandwich specimen for specified impact energy. In these experiments, the changes in the load-time history were captured by the Data Acquisition System (DAQ) incorporated with the drop weight impact test machine. At 5 J, the visual inspection method revealed that the absence of any visual damage on the impact facing sheet, when the different foam core sandwich panels were impacted. In response to the 5 J impact, the load-time (*L-t*) graphs display single mountain-like shape for all the sandwich panels. The [C/K/Foam core/K/C] and [H_2_/Foam core/H_2_] show the single primary load peaks, in a weak mode. As the visual inspection denotes the absence of either fiber breakage or matrix crackling in the sandwich panels, it indicates that the load is supported by elastic deformation thus leaving a trail of a single mount characteristic load-time (*L-t*) graph. The peak load-carrying capacity of the panel increases with the inter-ply and intra-ply hybrid structures of the sandwich panels. At 5 J, from the base sandwich panel with the carbon foam core sandwich [C_2_/Foam core/C_2_] layering sequence, a steady increase is observed in the peak load from 636.53 N to 802.58 N for the intra-ply carbon/Kevlar sandwich [H_2_/Foam core/H_2_]. A small dent is observed for the [H_2_/Foam core/H_2_] and [K_2_/Foam core/K_2_] panels indicating the core deformation. The maximum load for the impact event at 5 J was borne by inter-ply carbon/Kevlar sandwich [K/C/Foam core/C/K] with 1253.13 J, while the [C_2_/Foam core/C_2_] was the lowest with 636.53 N. The panels with intra-ply carbon/Kevlar sandwich [H_2_/Foam core/H_2_], inter-ply carbon/Kevlar sandwich [C/K/Foam core/K/C] and Kevlar face sheet foam core sandwich [K_2_/Foam core/K_2_] revealed the maximum load of 802.58 N, 847.03 N and 1225.74 N, respectively, as shown in Figure 12.

Very clearly, Figure 13 shows the characteristic load-time (*L-t*) graphs for the five combinations of the hybrid foam core sandwich panels, subjected to an impact load of 10 J. For the 10 J impact, the visual inspection reveals the damage to the impacted face sheet in three types of panels namely- carbon foam core sandwich [C_2_/Foam core/C_2_], the intra-ply carbon /Kevlar sandwich [H_2_/Foam core/H_2_] and the inter-ply carbon/Kevlar sandwich [C/K/Foam core/K/C]. The matrix cracks and fiber failure are clearly observable in these types of panels. The primary peak depicted by the load-time (*L-t*) graphs of [C_2_/Foam core/C_2_], [H_2_/Foam core/H_2_], [C/K/Foam core/K/C] panels indicate the damage on the impacted side face sheet with the primary peaks signifying the failure of the primary face sheets. At 10 J, the inter-ply carbon/Kevlar sandwich [K/C/Foam core/C/K] has the maximum load capacity of 2512.40 N and the [C_2_/Foam core/C_2_] has the least maximum load capacity with 962.25 N. The panels with intra-ply carbon/Kevlar sandwich [H_2_/Foam core/H_2_], inter-ply hybrid carbon/Kevlar sandwich [C/K/Foam core/K/C] and Kevlar-foam core sandwich [K_2_/Foam core/K_2_] have the maximum load of 1032.84 N, 1402.48 N and 1921.97 N, respectively, as shown in Figure 13. At 10 J impact, the inter-ply carbon/Kevlar sandwich [C/K/Foam core/K/C] and Kevlar foam core sandwich [K_2_/Foam core/K_2_] exhibited the parabolic curve which implies that the primary impacted side face sheet was not damaged during the impact event.

At 20 J, the primary and the secondary peaks in the load-time (*L-t*) graphs are prominent, as shown in Figure 14. Visible damage is observed on the impacted face sheet for the entire configurations of sandwich panels. The main damage to the impacted primary face sheet is caused by matrix cracking, fiber breakage and crushing of the core damage. The secondary peak begins to emerge in the curves, showing the contribution of the secondary non-impacted face sheets. At the 20 J impact energy, the inter-ply carbon/Kevlar sandwich [K/C/Foam core/C/K] has the maximum load capacity with 2744.53 N and the [C_2_/Foam core/C_2_] has the least maximum load capacity, 1082.87 N. The panels with intra-ply carbon/Kevlar sandwich [H_2_/Foam core/H_2_], the inter-ply carbon/Kevlar sandwich [C/K/Foam core/K/C] and Kevlar-foam core sandwich [K_2_/Foam core/K_2_] carried the maximum load of 1198.63 N, 2026.23 N and 2593.25 N, respectively, as displayed in Figure 14.

In fact, Figure 15 reveals the load-time (*L-t*) graphs of the various specimens impacted at 30 J. The secondary peak is distinctly present in all the variations of the foam core sandwich panels with the exception of Kevlar foam core sandwich [K_2_/Foam core/K_2_]. At 30 J impact energy, the inter-hybrid carbon/Kevlar sandwich [K/C/Foam core/C/K] shows the maximum load capacity of 3182.46 N while [C_2_/Foam core/C_2_] shows the least impact load capacity of 1112.27 N. The panels with the intra-ply carbon/Kevlar sandwich [H_2_/Foam core/H_2_], inter-hybrid carbon/Kevlar sandwich [C/K/Foam core/K/C] and Kevlar foam core sandwich [K_2_/Foam core/K_2_] had a maximum load of 1491.579 N, 2286.267 N and 3111.41 N, respectively.

By 40 J, the primary and the secondary peaks in the load-time (*L-t*) graphs are distinct and clearly evident. Visible damage is obvious on the impacted face sheet, of the entire set of sandwich panels, and damage to the secondary face sheets has been initiated. Through-the-thickness fracture is noted in the specimens of [C_2_/Foam core/C_2_] and [H_2_/Foam core/H_2_]. The major damage to the specimens has been caused by matrix cracking, fiber breakage and crushing of the core. The secondary peaks, which visibly emerge in the curves, reveals the contribution of the secondary non-impacted face sheets. At 40 J impact energy, the inter-ply carbon/Kevlar sandwich [K/C/Foam core/C/K] has the maximum load capacity of 3723.89 N, while the [C_2_/Foam core/C_2_] reveals the lowest maximum load capacity of 1368.16 N. The panels with the intra-ply carbon/Kevlar sandwich [H_2_/Foam core/H_2_], inter-hybrid carbon/Kevlar sandwich [C/K/Foam core/K/C] and Kevlar foam core sandwich [K_2_/Foam core/K_2_] bear a maximum load of 1548.31 N, 2829.67 N, and 3491.15 N, respectively, as displayed in Figure 16. The addition of the Kevlar layer has significantly improved the impact performance of the sandwich panels when compared with the [C_2_/Foam core/C_2_] panels [27].

### 4.2. Comparison of Peak Loads of Different Combinations of Foam Core Sandwich Panels 

**Figure 17:**
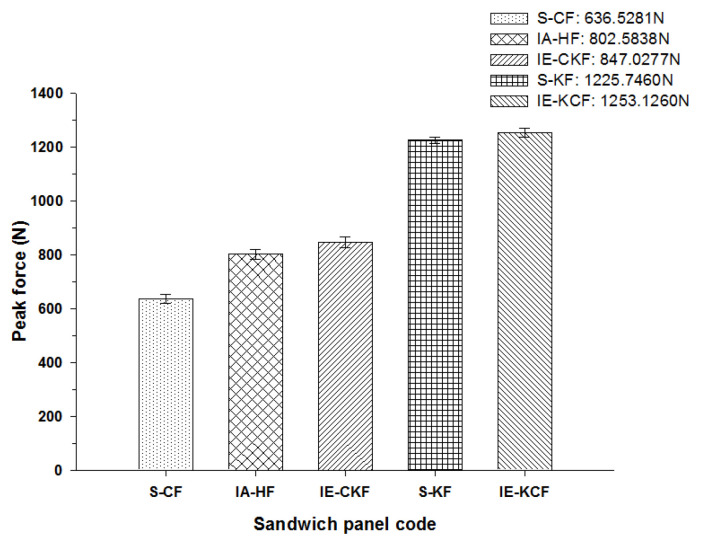
Comparison of peak force of different foam core sandwich specimens at 5 J.

**Figure 18:**
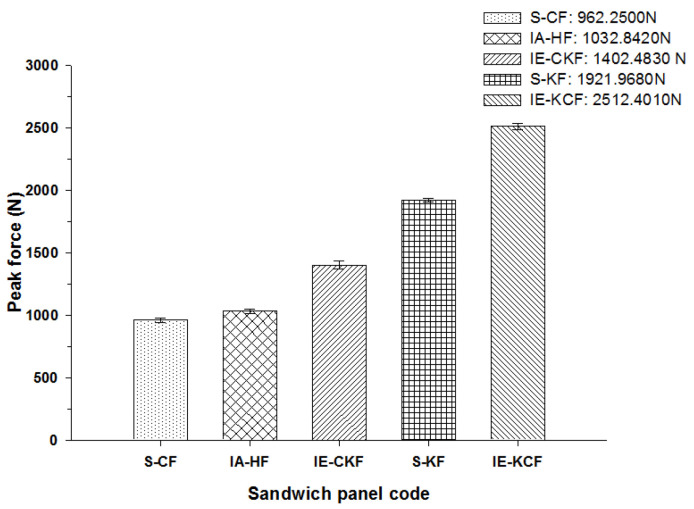
Comparison of peak force of different foam core sandwich specimens at 10 J.

**Figure 19:**
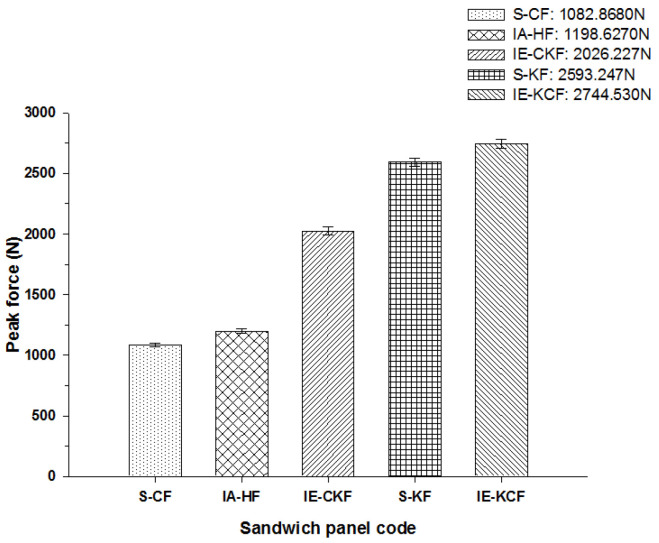
Comparison of peak force of different foam core sandwich specimens at 20 J.

**Figure 20:**
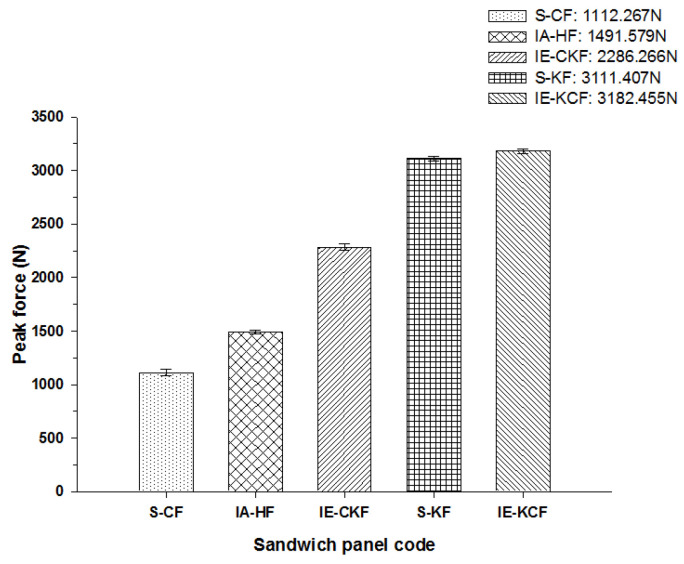
Comparison of peak force of different foam core sandwich specimens at 30 J.

**Figure 21:**
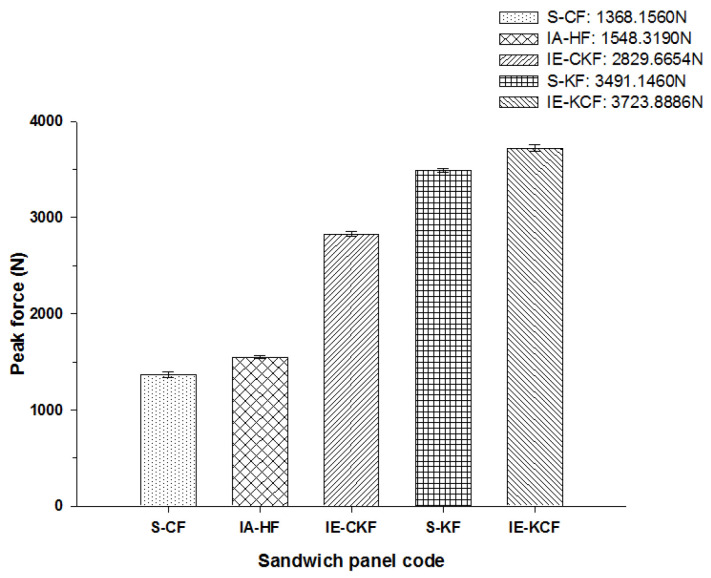
Comparison of peak force of different foam core sandwich specimens at 40 J.

The peak load comparison bar charts illustrated in Figure 17, Figure 18, Figure 19, Figure 20 and Figure 21 clearly describe the peak load variations, at specific incident energies. The peak load defines the maximum load that the panel exerts against the applied impact force [37,38,42]. This graphical representation displays the variations in the peak load with respect to the various sandwich panels represented in Table 2. At the 5 J impact energy, the intra-ply carbon/Kevlar sandwich [H_2_/Foam core/H_2_] shows an increase in the peak load capacity of 26.08% when compared with the carbon face sheet foam core sandwich [C_2_/Foam core/C_2_] panel, and the inter-ply carbon/Kevlar sandwich [K/C/Foam core/C/K] shows a rise of 2.20% more than the Kevlar foam core sandwich [K_2_/Foam core/K_2_] panels as depicted in Figure 17. The peak load for the inter-ply hybrid foam core sandwich panels [C/K/Foam core/K/C] and [K/C/Foam core/C/K] panels are 52.72% and 56.13%, respectively, when compared with the intra-ply carbon/Kevlar sandwich [H_2_/Foam core/H_2_] panel, respectively. At 10 J impact energy, the intra-ply carbon/Kevlar sandwich [H_2_/Foam core/H_2_] shows an increase of 7.30% in the peak load capacity when compared with carbon face sheet foam core sandwich [C_2_/Foam core/C_2_] panel; and the inter-ply carbon/Kevlar sandwich [K/C/Foam core/C/K] shows an increase of 30.7% increase above the Kevlar foam core sandwich [K_2_/Foam core/K_2_] panels, as displayed in Figure 18. At 10 J, the impact energy peak load for the intra-ply hybrid foam core sandwich [K/C/Foam core/C/K] panel is 79.13%, when compared with the [C/K/Foam core/K/C]. At the 20 J impact energy, the intra-ply carbon/Kevlar sandwich [H_2_/Foam core/H_2_] shows a peak load capacity increase of 10.69% when compared with the carbon face sheet foam core sandwich [C_2_/Foam core/C_2_] panel and the inter-ply carbon/Kevlar sandwich [K/C/Foam core/C/K] reveals an increase of 5.83% more than the Kevlar foam core sandwich [K_2_/Foam core/K_2_] panels, as shown in Figure 19. At 20 J, the impact energy peak load for the inter-ply hybrid foam core sandwich panel [K/C/Foam core/C/K] panel is 35.45% more, when compared with the [C/K/Foam core/K/C]. At 30 J impact energy, the intra-ply carbon/Kevlar sandwich [H_2_/Foam core/H_2_] shows a peak load capacity rise of 34.10% when compared with the monolithic carbon face sheet foam core sandwich [C_2_/Foam core/C_2_] panel; and inter-ply carbon/Kevlar sandwich [K/C/Foam core/C/K] reveals an increase of 2.28% more than the Kevlar foam core sandwich [K_2_/Foam core/K_2_] panel, as shown in Figure 20. For the 30 J the impact energy, the peak load for the inter-ply hybrid foam core sandwich panel [K/C/Foam core/C/K] panel is 39.20% higher when compared with [C/K/Foam core/K/C]. At 40 J impact energy the intra-ply carbon/Kevlar sandwich [H_2_/Foam core/H_2_] shows a peak load capacity increase of 13.17% when compared with the carbon face sheet foam core sandwich [C_2_/Foam core/C_2_] panel; and the inter-ply carbon/Kevlar sandwich [K/C/Foam core/C/K] has an increase of 6.67% more than the Kevlar foam core sandwich [K_2_/Foam core/K_2_] panels as shown in Figure 21. At 40 J, the impact energy peak load for the intra-ply hybrid foam core sandwich [K/C/Foam core/C/K] panel is 31.57% higher when compared with the [C/K/Foam core/K/C].

### 4.3. Post-Impact Inspection of Panels

**Figure 22:**
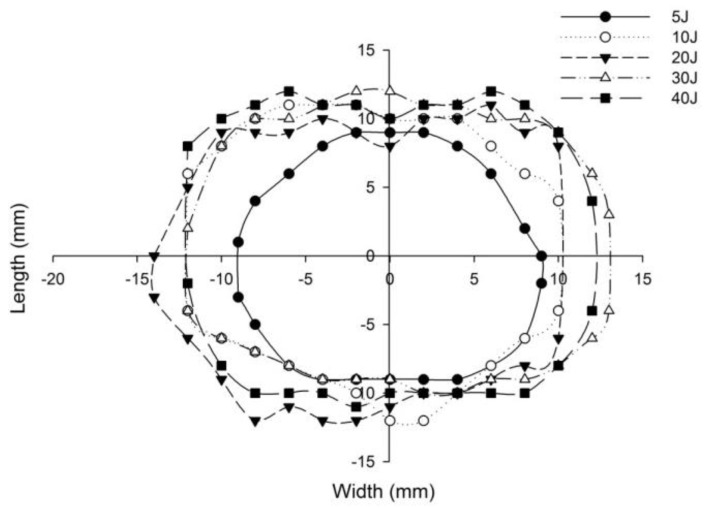
Graph illustrating incident face sheet damage area of [C_2_/Foam core/C_2_] sandwich specimens corresponding to different impact energy levels.

**Figure 23:**
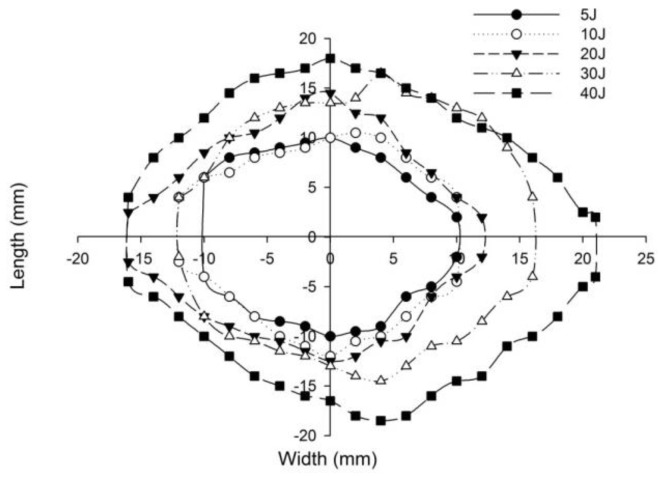
Graph illustrating incident face sheet damage area of [K_2_/Foam core/K_2_] sandwich specimens corresponding to different impact energy levels.

**Figure 24:**
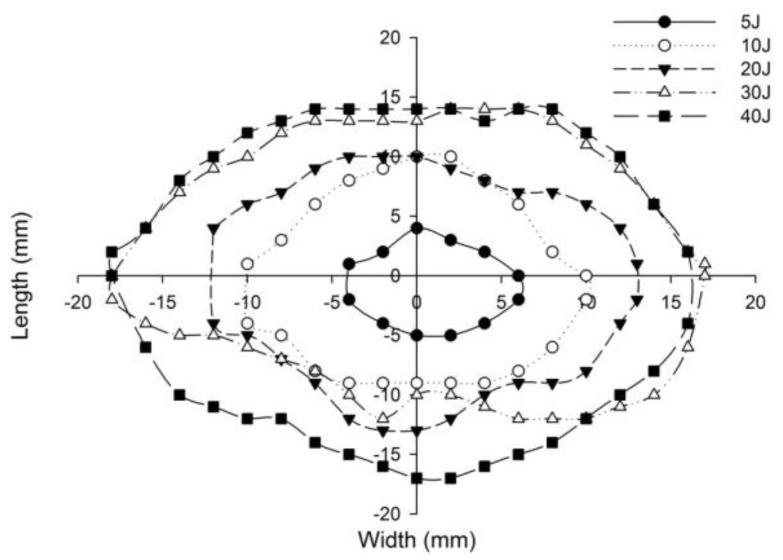
Graph illustrating incident face sheet damage area of [C/K/Foam core/K/C] sandwich specimens corresponding to different impact energy levels.

**Figure 25:**
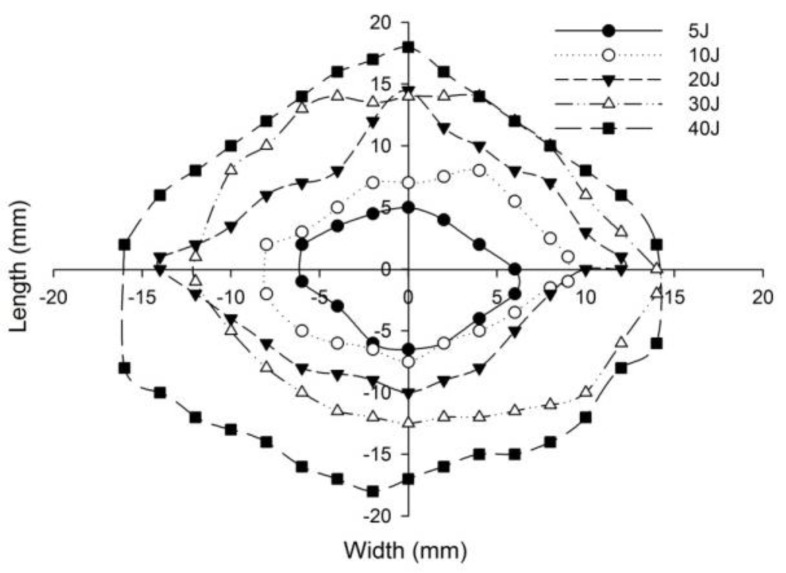
Graph illustrating incident face sheet damage area of [K/C/Foam core/C/K] sandwich specimens corresponding to different impact energy levels.

**Figure 26:**
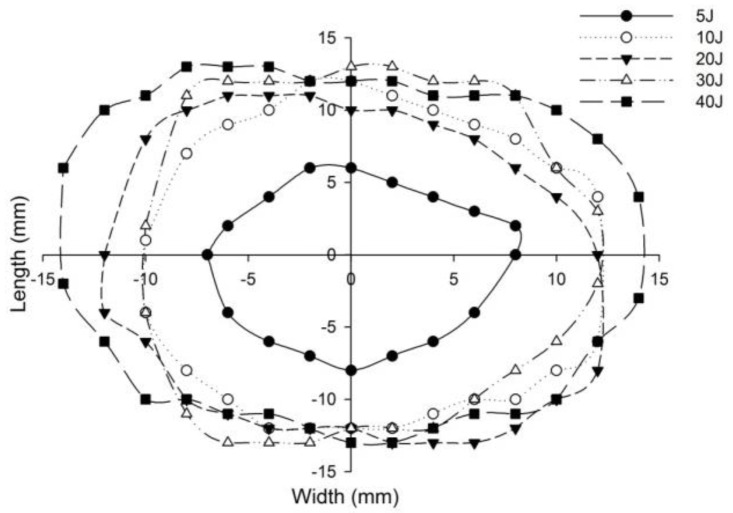
Graph illustrating incident face sheet damage area of [H_2_/Foam core/H_2_] sandwich specimens corresponding to different impact energy levels.

The post-impact inspections on the sandwich panels focusing on the damage to the incident face sheets of different sandwich panels, impacted with different energy levels, are shown from Figure 22, Figure 23, Figure 24, Figure 25 and Figure 26. The increase in the damage of the sandwich panels, under different impact energy levels, is plotted along the face-wise direction. The size of delaminated damage area with respect to the incident face sheet, has been plotted for each specimen and superimposed for the different energy levels. For the carbon monolithic face sheet sandwich [C_2_/Foam core/C_2_] panels, the incident face sheet damage area is depicted by double dumbbell shapes and an increase in the damage area is displayed in response to the increase in the impact energy. In Figure 23, the Kevlar monolithic face sheet sandwich panels [K_2_/Foam core/K_2_] reveal the damage areas in rhombus-like shapes. The incident face sheet damage areas of the [C/K/Foam core/K/C] sandwich specimens resemble an oval shape, as seen in Figure 24. In Figure 25, the inter-ply hybrid foam core sandwich panel [K/C/Foam core/C/K] sandwich specimens display the damage area, which closely resembles an isosceles rhombus shape. In the intra-ply carbon/Kevlar sandwich panel [H_2_/Foam core/H_2_], the damage area appears as a rectangular shape, as shown in Figure 26.

The length of the surface damage in the incident face sheet is measured along the two mutually perpendicular directions as illustrated in Figure 26, and the average surface damage length (d) is tabulated in Table 5. The increase in the surface damage in terms of the increase in the impact energy is clearly shown in the tabulation. Considering the inter-ply carbon/Kevlar sandwich panels the [K/C/Foam core/C/K] show a decrease of 17.98%, 18.69%, 6.63%, 8.86% and 6.27%, respectively, in the average length of the surface damage incident face sheet against the [C/K/Foam core/C/K] panels in response to impact energies of 5 J, 10 J, 20 J, 30 J and 40 J.

**Figure 27:**
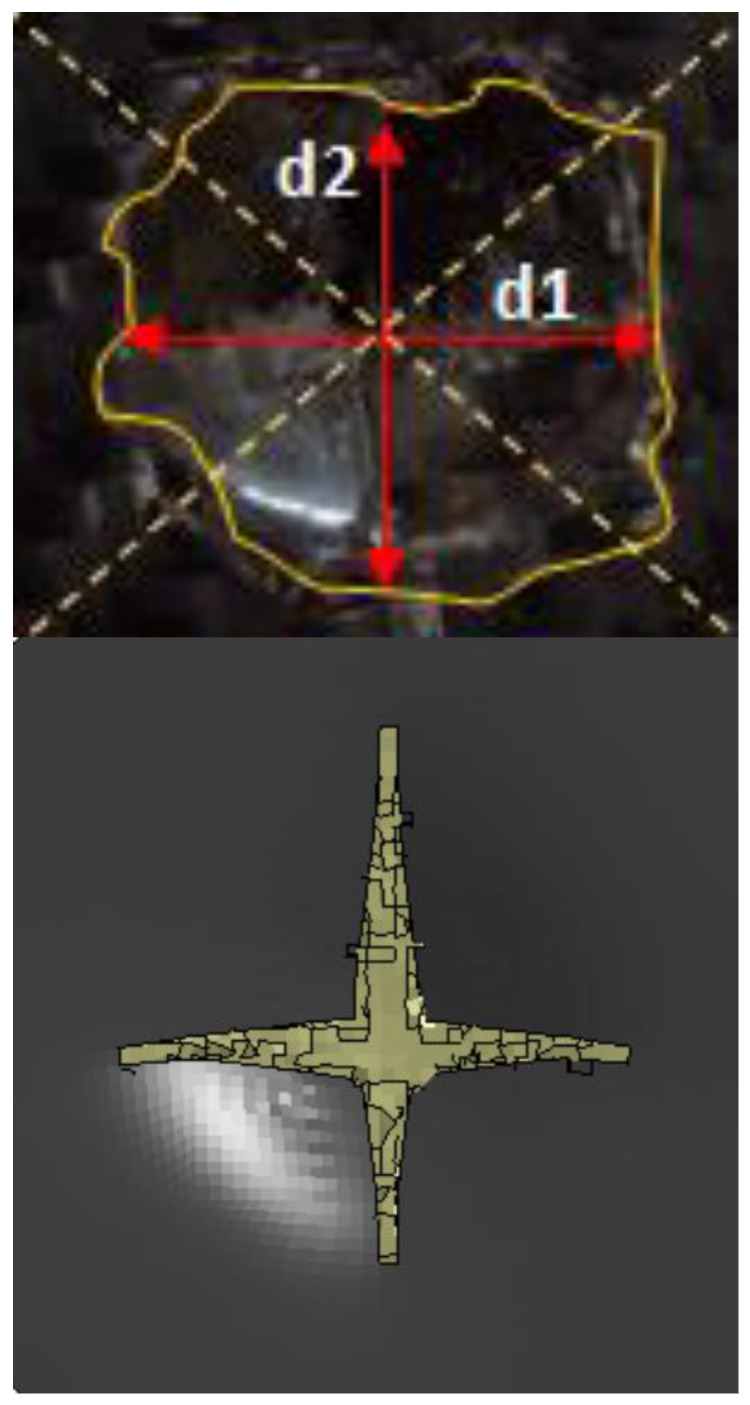
Photograph illustrating the measurement of length of the surface damage d1 and d2 on incident (primary) face sheet of [C_2_/Foam core/C_2_] with the numerical simulation result.

**Figure 28:**
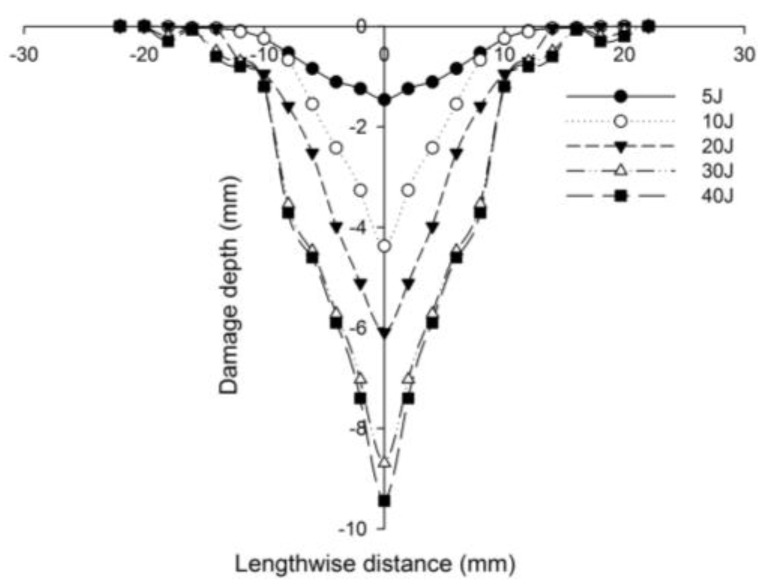
Residual dent depth with respect to the lengthwise distance of [C_2_/Foam core/C_2_] sandwich specimens impacted with different energy levels.

**Figure 29:**
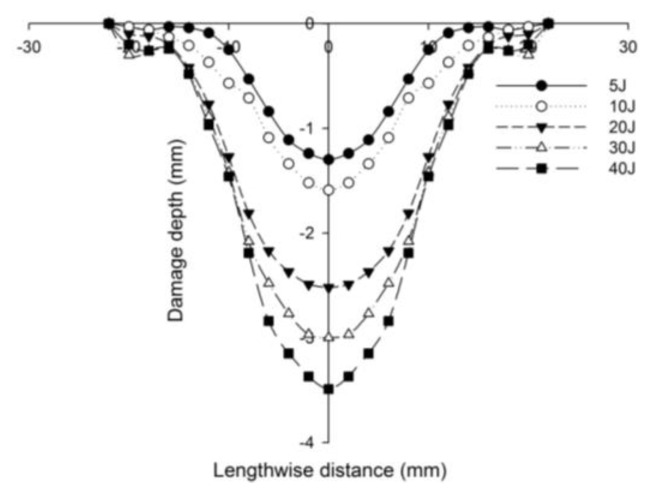
Residual dent depth with respect to the lengthwise distance of [K_2_/Foam core/K_2_] sandwich specimens impacted with different energy levels.

**Figure 30:**
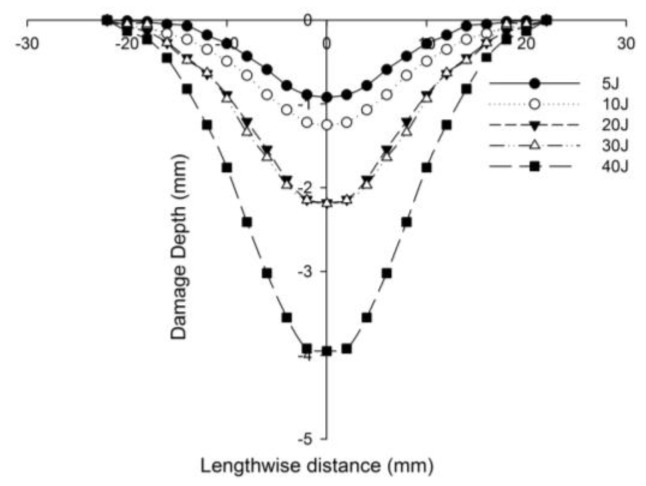
Residual dent depth with respect to the lengthwise distance of [C/K/Foam core/K/C] sandwich specimens impacted with different energy levels.

**Figure 31:**
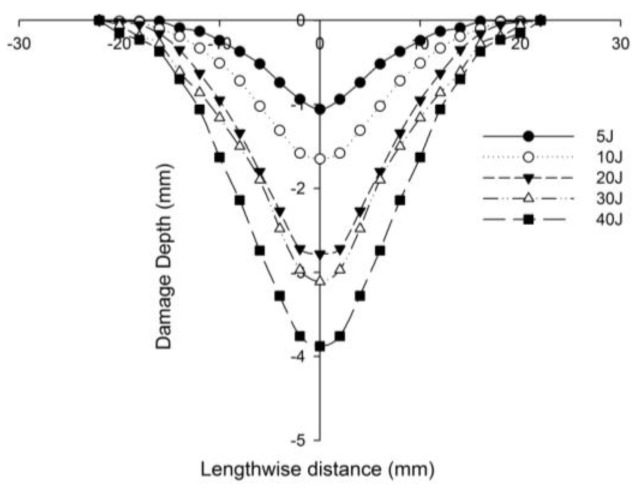
Residual dent depth with respect to the lengthwise distance of [K/C/Foam core/C/K] sandwich specimens impacted with different energy levels.

**Figure 32:**
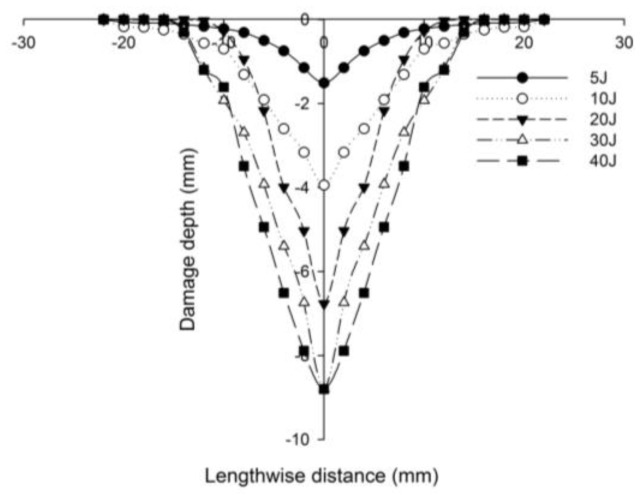
Residual dent depth with respect to the lengthwise distance of [H_2_/Foam core/H_2_] sandwich specimens impacted with different energy levels.

**Figure 33:**
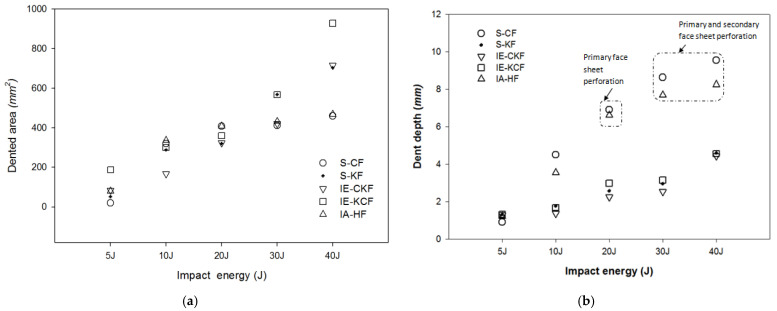
(**a**) Dented area on the top facing sheet of various sandwich panels. (**b**) Dent depth of the top facing sheet of sandwich panels with respect to the different energy levels.

**Figure 34:**
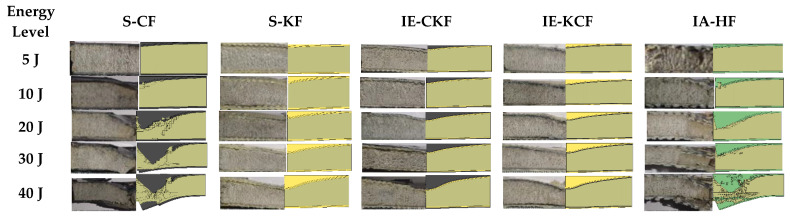
Comparison of experimental and numerical failure modes of sandwich panels subjected to varying levels of impact energy.

Figure 27 compares the numerical simulation result with images exhibiting the measurement of the length of the surface damage on the incident (main) face sheet of [C_2_/Foam core/C_2_].The residual dent depths, with respect to the lengthwise distance of the various sandwich specimens, namely the [C_2_/Foam core/C_2_]; [K_2_/Foam core/K_2_]; inter-ply face sheets [C/K/Foam core/K/C]; [K/C/Foam core/C/K]; intra-ply face sheets [H_2_/Foam core/H_2_], impacted with different energy levels, were measured using a high-precision micrometer gauge, and the results are displayed from Figure 28, Figure 29, Figure 30, Figure 31 and Figure 32. In fact, Figure 33 shows the variations in the dented area on the top facing sheet of the sandwich panels, in response to the different energy levels, and the dent depth of the top facing sheet of the sandwich panels, in response to the different impact energies. Figure 34 shows the through-thickness comparison of the numerical simulations with the experimental results. The maximum load carrying capacity of the numerical simulation is compared with the experimental results in Table 6. The numerical simulation using the commercially available LS-DYNA software shows excellent correlation with the experiments, with deviations within 10% in all the various sandwich structures and various impact energy levels. When the Divinycell H 80 foam fails, it is due to a pure core shear failure; otherwise, it is due to a mixture of shear plug failures. A constitutive model that takes into account the foam orthotropy and coupling between all stress components is required for accurate numerical prediction. Rajaneesh [57] investigated the failure pattern of Divinycell H 80 foam in depth, and the numerical results obtained showed a similar failure pattern.

**Figure 35:**
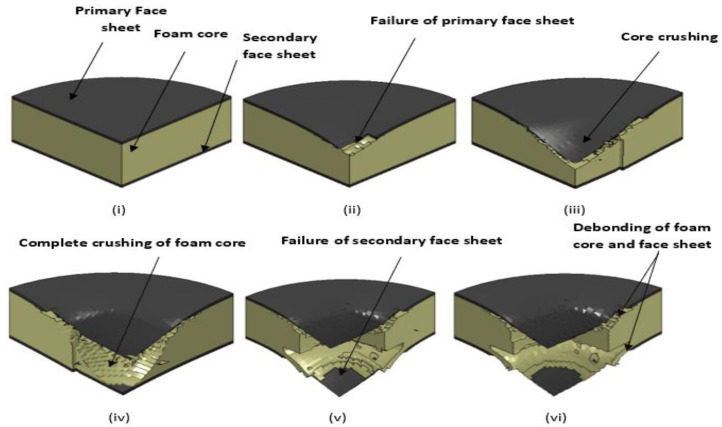
The progression of damage to the face sheet and foam core at various stages of impact (**i**) before the impactor contacting the primary face sheet (**ii**) failure of primary face sheet (**iii**) core crushing (**iv**) complete crushing of the core (**v**) failure of the secondary face sheet (**vi**) debonding of foam.

**Figure 36:**
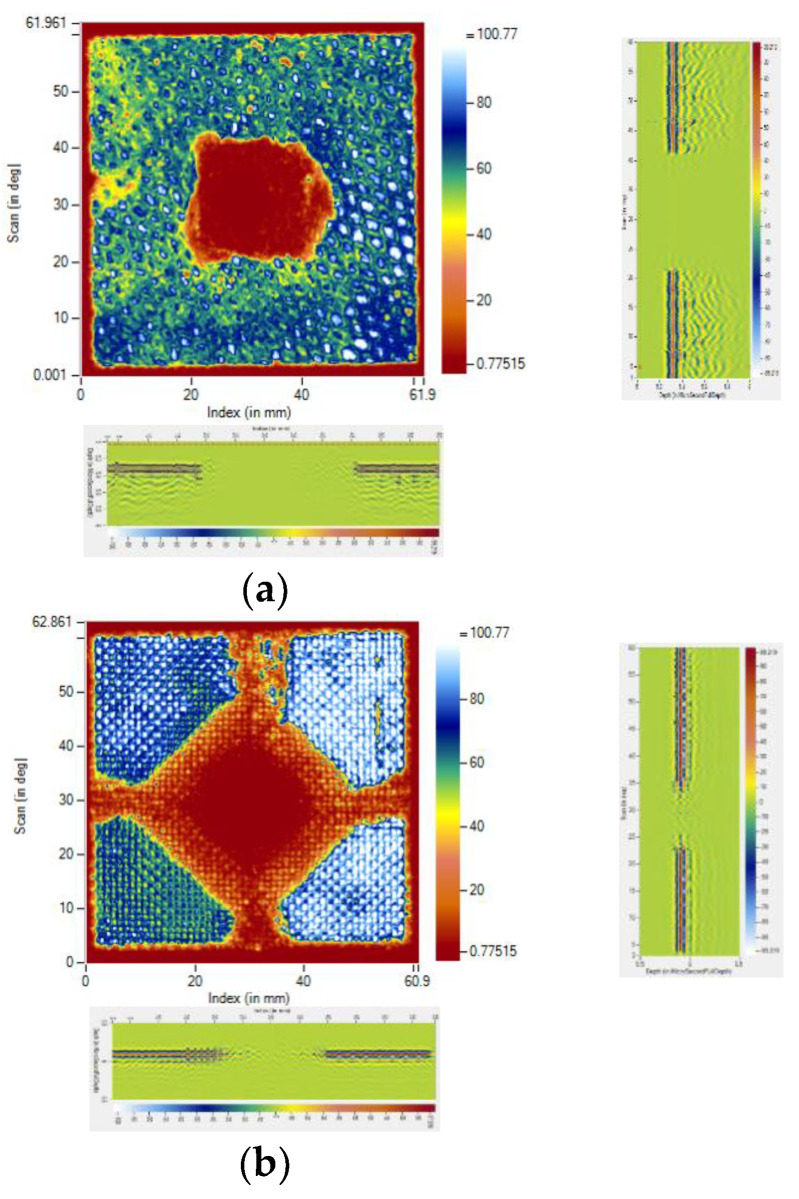

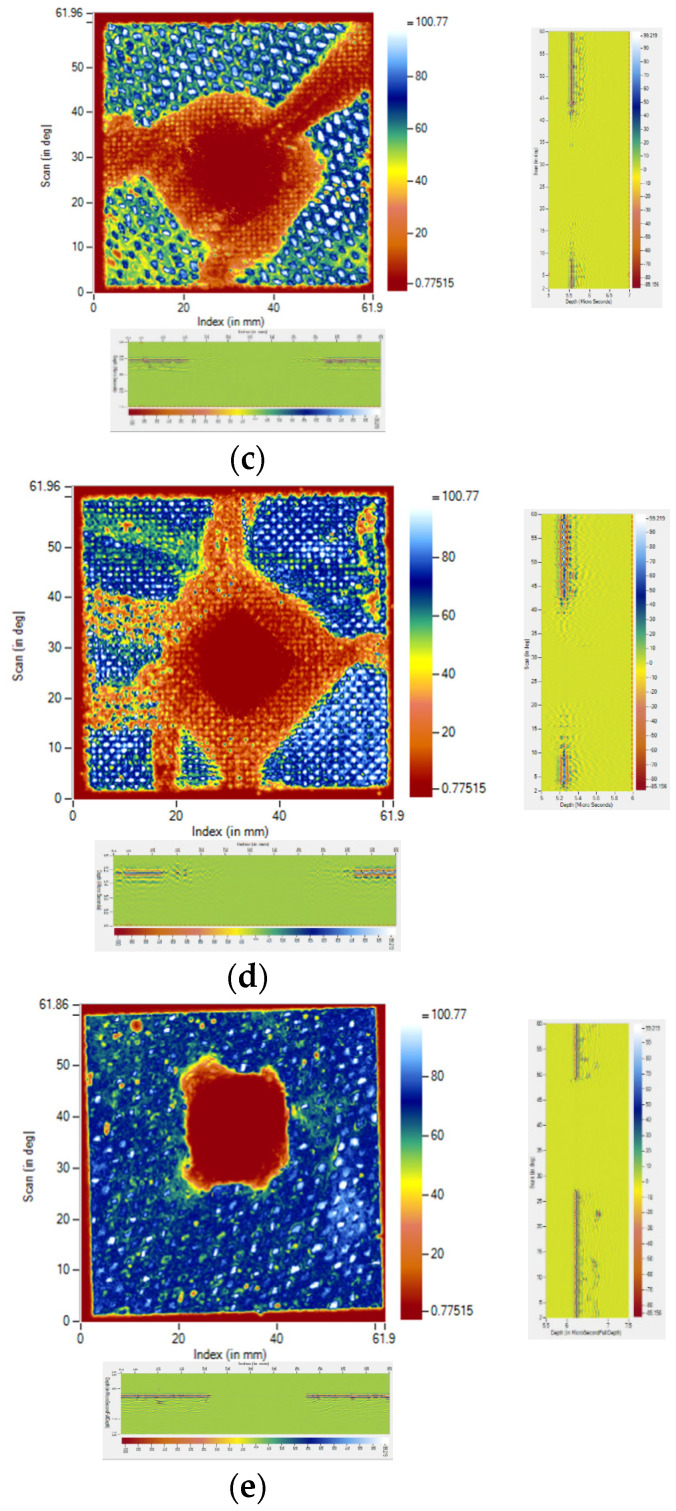
B-Scan, C-Scan and D-Scan images for (**a**) [C_2_/Foam core/C_2_]; (**b**) [K_2_/Foam core/K_2_]; (**c**) [C/K/Foam core/K/C]; (**d**) [K/C/Foam core/C/K]; (**e**) [H_2_/Foam core/H_2_] specimens impacted by 40 J.

**Figure 37:**
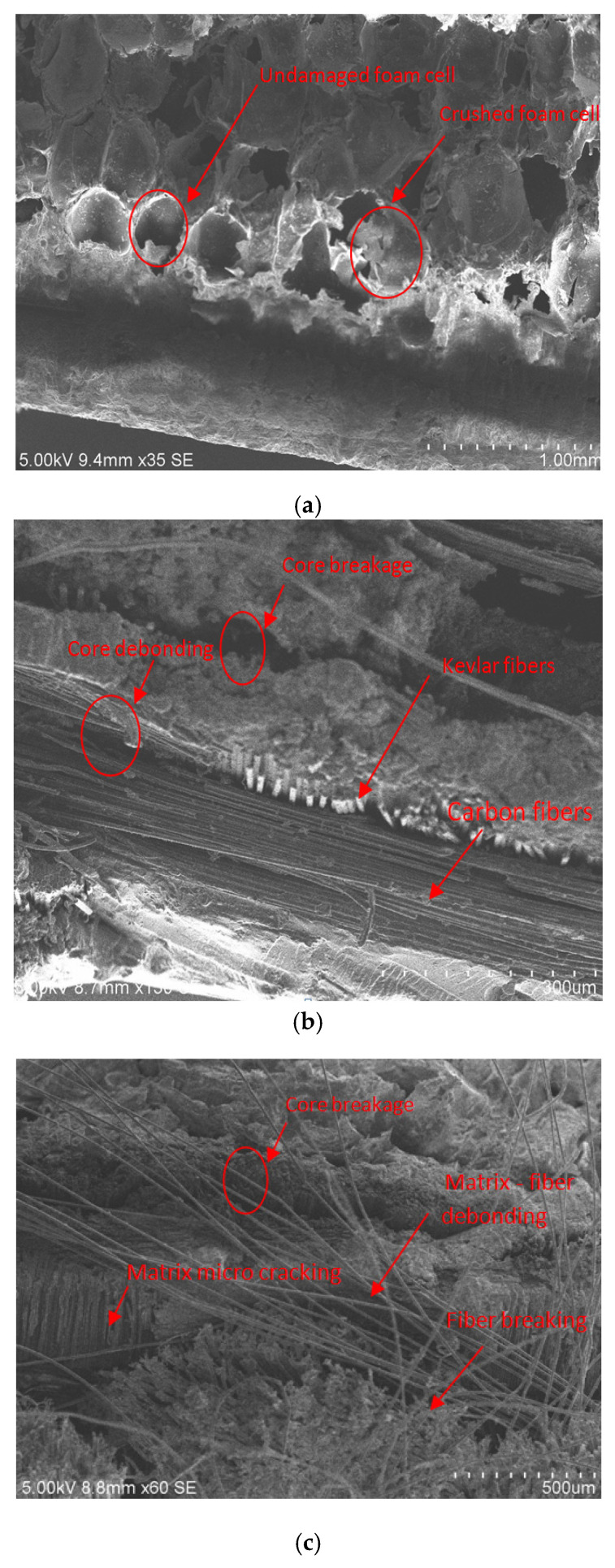

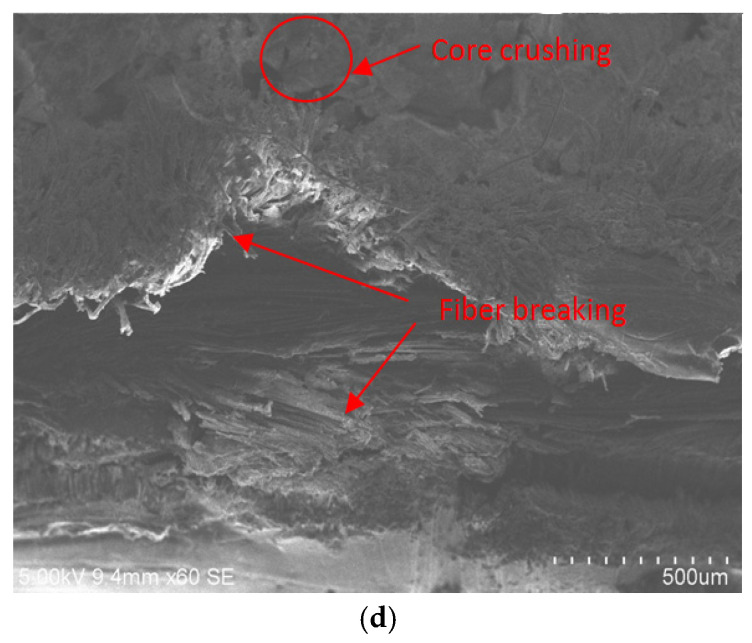
Characteristic Scanning Electron Microscopy (SEM) microstructure in the region of low-velocity impact of sandwich specimens: (**a**) impacted foam core, (**b**) interface between the foam and face sheet, (**c**) impacted face sheet region, (**d**) debonding zone.

Figure 35 illustrates the impact damage progression to the face sheet and foam core in the LS-DYNA simulation at various time steps. Figure 36 shows the ultrasonic water immersion scans (B-Scan, C-Scan and D-Scan images) for the [C_2_/Foam core/C_2_]; [K_2_/Foam core/K_2_]; [C/K/Foam core/K/C]; [K/C/Foam core/C/K]; and [H_2_/Foam core/H_2_] specimens, impacted with the 40 J impact energy. The characteristic Scanning Electron Microscopy (SEM) was used to probe the failure mechanisms in the regions of the low-velocity impact of the sandwich specimens. In the SEM scan, the impacted foam core, interface between the foam and face sheet, impacted face sheet region, and debonding zone as seen in Figure 37, were carefully studied. In the impact of inter-ply sandwich panels with a Kevlar face sheet as the outer layer, the load is transferred to the ductile layer of Kevlar initially; thus, the dent depth was measured less when compared with other panels [53].

## 5. Conclusions

In this paper the low-velocity impact response of foam core sandwich panels with the inter-ply and intra-ply carbon/Kevlar hybrid face sheets are extensively investigated and compared with the monolithic carbon and Kevlar sandwich panels. From each low-velocity impact test conducted, the visual evaluation of the specimen, as well as the ultrasonic water immersion C-Scan and SEM scan were done, and the comprehensive impact behavior was observed. The following conclusions can be quantified:

- The low-velocity impact-resistant properties of the carbon fiber face sheet sandwich composites are improved with the addition of the intra-ply and inter-play hybrid Kevlar face sheets. The addition of a Kevlar layer to the face sheet improves the peak load carrying capacity of foam core sandwich panels. The inter-ply carbon/Kevlar sandwich panel shows a considerable increase of peak load when the Kevlar-ply is placed as the outer lamina in the incident face sheet. The visual inspection of the drop weight impacted specimens demonstrates the diverse damage patterns.
- The load curve for the sandwich specimens demonstrates a peak load with sudden fluctuations in the load, which denotes the failure of the incident face sheet; this is followed by a plateau region which represents the time required for the crushing of the core. The secondary peak in the load-time curve illustrates the failure of the secondary face sheet, which completes the perforation of the sandwich panel.
- The inter-ply Kevlar/carbon foam core sandwich with the Kevlar layer towards the other surface displayed a reduced length of the surface damage out of the non-perforated panels.
- The results reveal that at 30 J impact energy the intra-ply carbon/Kevlar sandwich panel displayed a peak load capacity increase of 34.10% when compared with monolithic carbon face sheet foam core sandwich panel, and at 40 J impact energy, the peak load carrying capacity of intra-ply hybrid foam core sandwich panel with Kevlar towards the outer surface of the panel is 31.57% higher when compared with the sandwich panel with carbon towards the outer surface.
- The maximum load predicted by numerical models is very close to that measured experimentally. Within a 10% margin of error, the maximum load carrying capacity of various panels was predicted.
- By visual inspection and the SEM scan, the primary damage mechanisms of the sandwich panels have been identified as fiber breakage, matrix failure, debonding of the core and the face sheet and core crushing. A small increase in the dented area is evident, but the dent depth has a significant decrease for the inter-ply hybrid sandwich panels. The water immersion C-Scan revealed the discontinuity produced by the low-velocity impact on the face sheets and the interface between the face sheet laminas. The intra-ply hybrid panels have greater load carrying capacity than the monolithic sandwich panels when subjected to low-velocity impact.

## Figures and Tables

**Table 1:** Mechanical properties of Divinycell-‘H’-80 sandwich core material manufactured by Diab Group Inc. [49].

Property	Value
Density (kg/m^3^)	80
Compressive Strength (Mpa)	1.28
Compressive Modulus (Mpa)	85
Tensile Strength (Mpa)	2.4
Tensile Modulus (Mpa)	80
Shear Strength (Mpa)	1.05
Shear Modulus (Mpa)	25
Shear Strain (%)	30

**Table 2:** Woven fabric properties provided in data sheet by the manufacturer [50].

Property	Cabron	Kevlar	Intra-Ply Hybrid
Weave pattern	2 × 2 Twill weave	Plain weave	2 × 2 Twill weave
Area density (g/m^3^)	193	170	186
Thickness (mm)	0.305	0.298	0.305
Rows per inch	12.5 × 12.5	15 × 15	12.5 × 12.5

**Table 3:** Properties of GY 257 modified liquid epoxy resin system resin manufactured by Huntsman Inc. [51].

Property	Value
Appearance	Clear
Epoxy value (eq/Kg)	5.15–5.45
Weight per epoxide (g/eq)	185–195
Viscosity at 25 °C (mPa.s)	525–725
Density at 25 °C (g/mm^3^)	1.15 × 10^−6^
Flash point	≥140 °C

**Table 4:** Configuration of specimens with hybrid structure notation and layer sequence.

Specimen Notation	Hybrid Structure	Layer Sequence	Average Thickness (mm)	Diagram
S-CF	Carbon-foam core sandwich	[C_2_/Foam core/C_2_]	12.40	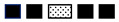
S-KF	Kevlar-foam core sandwich	[K_2_/Foam core/K_2_]	12.30	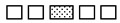
IE-CKF	Inter-hybrid carbon/Kevlar sandwich	[C/K/Foam core/K/C]	12.36	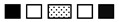
IE-KCF	Inter-hybrid carbon/Kevlar sandwich	[K/C/Foam core/C/K]	12.35	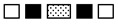
IA-HF	Intra-hybrid carbon/Kevlar sandwich	[H2/Foam core/H2]	12.37	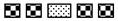

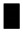
-Carbon woven fabric; 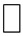-Kevlar woven fabric; 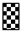-Intra-hybrid carbon/Kevlar; 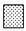-Foam core.

**Table 5:** Summary of incident face sheet surface damage length of various sandwich panels.

Sandwich Panel Code	Sandwich Structure	Energy Level (J)	Length of the Surface Damage (Incident Face Sheet) d1 (mm)	Length of the Surface Damage (Incident Face Sheet) d2 (mm)	Average Length of the Surface Damage (Incident Face Sheet) d (mm)
S-CF	Carbon-foam core sandwich	5	17.00	17.04	17.02
		10	24.21	19.06	21.64 ^1^
		20	22.42	21.91	22.17 ^2^
		30	24.09	20.90	22.50 ^2^
		40	24.36	20.98	22.67 ^2^
S-KF	Kevlar-foam core sandwich	5	20.43	20.01	20.22
		10	22.39	21.90	22.15
		20	28.47	26.42	27.44
		30	28.44	27.00	27.72
		40	37.16	34.46	35.81
IE-CKF	Inter-ply Carbon/Kevlar foam core sandwich	5	12.04	11.20	11.62
		10	20.04	19.02	19.53
		20	25.22	22.98	24.10
		30	34.83	22.95	28.89
		40	34.30	31.04	32.67
IE-KCF	Inter-ply Kevlar/carbon foam core sandwich	5	10.10	8.95	9.53
		10	17.24	14.51	15.87
		20	22.40	22.60	22.50
		30	26.13	26.52	26.33
		40	30.28	30.96	30.62
IA-HF	Intra-ply carbon/Kevlar foam core sandwich	5	14.92	13.92	14.42
		10	23.98	21.98	22.98
		20	22.32	23.91	23.11 ^1^
		30	22.43	24.80	23.61 ^2^
		40	24.40	24.97	24.68 ^2^

^1^ Primary face sheet perforation; ^2^ Primary and Secondary face sheet perforation.

**Table 6:** Maximum load comparison between numerical and experimental results.

Sandwich Panel Code	Energy Level (J)	Experimental Maximum Load (N)	Numerical Maximum Load (N)	% Deviation
S-CF	5	636.5	658.3	3.42
	10	962.3	1008.3	4.79
	20	1082.9	1180.2	8.99
	30	1112.3	1222.6	9.92
	40	1368.2	1470.4	7.47
S-KF	5	802.6	862.3	7.44
	10	1922.0	2110.8	9.82
	20	2593.2	2682.9	3.46
	30	3111.4	3283.6	5.53
	40	3491.1	3828.4	9.66
IE-CKF	5	847.0	924.3	9.12
	10	1402.5	1536.6	9.56
	20	2026.2	2184.8	7.83
	30	2286.3	2492.8	9.03
	40	2829.7	2968.6	4.91
IE-KCF	5	1225.7	1328.6	8.39
	10	2512.4	2758.1	9.78
	20	2744.5	3018.3	9.98
	30	3182.5	3488.6	9.62
	40	3723.9	3883.2	4.28
IA-HF	5	802.6	872.3	8.69
	10	1032.8	1127.5	9.16
	20	1198.6	1302.5	8.67
	30	1491.6	1634.3	9.57
	40	1548.3	1690.2	9.16

## Data Availability

Data sharing not applicable.
